# Adiponectin Enhances B-Cell Proliferation and Differentiation *via* Activation of Akt1/STAT3 and Exacerbates Collagen-Induced Arthritis

**DOI:** 10.3389/fimmu.2021.626310

**Published:** 2021-03-18

**Authors:** Nan Che, Xiaoxuan Sun, Lei Gu, Xiaohui Wang, Jingjing Shi, Yi Sun, Lingxiao Xu, Rui Liu, Junke Wang, Fengyi Zhu, Na Peng, Fan Xiao, Dajun Hu, Liwei Lu, Wen Qiu, Miaojia Zhang

**Affiliations:** ^1^Department of Rheumatology, The First Affiliated Hospital of Nanjing Medical University, Nanjing, China; ^2^Department of Pathology, Shenzhen Institute of Research and Innovation, The University of Hong Kong, Hong Kong, China; ^3^Chongqing International Institute for Immunology, Hong Kong, China; ^4^Clinical Medical Science of the First Clinical Medical College, Nanjing Medical University, Nanjing, China; ^5^Department of Rheumatology and Nephrology, The Second People's Hospital of China Three Gorges University, Yichang, China; ^6^Department of Immunology, Key Laboratory of Immunological Environment and Disease, Nanjing Medical University, Nanjing, China

**Keywords:** adiponectin, B cells, proliferation, differentiation, collagen-induced arthritis

## Abstract

Although B cells have been shown to contribute to the pathogenesis of rheumatoid arthritis (RA), the precise role of B cells in RA needs to be explored further. Our previous studies have revealed that adiponectin (AD) is expressed at high levels in inflamed synovial joint tissues, and its expression is closely correlated with progressive bone erosion in patients with RA. In this study, we investigated the possible role of AD in B cell proliferation and differentiation. We found that AD stimulation could induce B cell proliferation and differentiation in cell culture. Notably, local intraarticular injection of AD promoted B cell expansion in joint tissues and exacerbated arthritis in mice with collagen-induced arthritis (CIA). Mechanistically, AD induced the activation of PI3K/Akt1 and STAT3 and promoted the proliferation and differentiation of B cells. Moreover, STAT3 bound to the promoter of the Blimp-1 gene, upregulated Blimp-1 expression at the transcriptional level, and promoted B cell differentiation. Collectively, we observed that AD exacerbated CIA by enhancing B cell proliferation and differentiation mediated by the PI3K/Akt1/STAT3 axis.

## Introduction

Rheumatoid arthritis (RA) is a common rheumatic disease characterized by chronic joint inflammation, which leads to cartilage and bone damage and progressive disability (1, 2). Owing to the combined effects of environmental and genetic factors, RA pathogenesis has become more complex with time. Despite intensive efforts focused on the development of novel therapies for preventing RA progression, the disease cannot be treated in a proportion of patients using current synthetic or biological disease-modifying anti-rheumatic drug therapy (3). Therefore, the identification of novel targets for RA therapy remains challenging.

B cells play important roles in RA pathogenesis. B cell-depletion therapy with rituximab significantly reduced the clinical symptoms and inflammation observed in RA and also prevented the progression of joint damage by increasing bone formation and decreasing bone resorption (4–6). Although B cells have been shown to contribute to RA pathogenesis through autoantibody production, antigen presentation, and CD4^+^ T cell activation, the precise role of B cells in RA pathogenesis needs to be explored further.

Increasing evidence has shown that adipokines, including adiponectin (AD), leptin, visfatin, and resistin, produced by white adipose tissues, play important roles in regulating immune responses and inflammatory processes (7–11). Findings from recent clinical studies have suggested that the serum AD levels in patients with RA are related to disease progression, implying that AD might contribute to RA pathogenesis (12). In our previous studies, we have demonstrated that AD is expressed at high levels in the inflamed synovial joint tissues, and AD expression is closely correlated with progressive bone erosion in patients with RA (13, 14). However, the effects of AD on B cell proliferation and differentiation remain unclear.

It is well-known that phosphoinositide-3-kinase (PI3K) is an important upstream regulator of Akt1, and the PI3K/Akt1 signaling pathway plays a pivotal role in B cell proliferation and differentiation (15). Signal transducer and activator of transcription 3 (STAT3) is a member of the STAT family. STAT3 activation is also known to contribute to B cell proliferation and differentiation (16). Notably, Diehl et al. (17) have reported that STAT3-mediated upregulation of Blimp-1 (a key B-cell differentiation regulator) is coordinated with BCL6 downregulation to control human B cell differentiation to plasma cells. Additionally, AD was shown to be capable of activating PI3K/Akt1 and STAT3 in some cell types, such as the epithelial cells and the liver cancer cells (18–20). The roles of AD in PI3K/Akt1 and STAT3 activation as well as in B cell proliferation and differentiation, remain uninvestigated.

In the present study, we determined the effects of AD on B cell proliferation and differentiation both in culture and in mice with collagen-induced arthritis (CIA). We also studied PI3K/Akt1 and STAT3 activation and its role in AD-induced B cell proliferation and differentiation. Additionally, the effects of PI3K/Akt1/STAT3 activation on B lymphocyte-induced maturation protein-1 (Blimp-1), a key B-cell differentiation regulator, were also examined. Collectively, our findings indicated that AD exacerbates CIA by enhancing B cell proliferation and differentiation mediated by the PI3K/Akt1/STAT3 axis.

## Methods

### Reagents

Monoclonal antibodies against human β-actin (4970), ERK1/2 (4695), phospho-ERK1/2^Thr202/Tyr204^ (p-ERK1/2, 4370), PI3K p110δ (34050), Akt1 (2938), phospho-Akt1^Thr308^ (p-Akt1^Thr308^, 4056), phospho-Akt1^Ser473^ (p-Akt1^Ser473^, 9018), STAT3 (9139), phospho-STAT3^Tyr705^ (p-STAT3, 9145), and Egr-1 (4153), along with HRP-conjugated anti-rabbit immunoglobulin G (IgG) (7074) and HRP-conjugated anti-mouse IgG (7076) were purchased from Cell Signaling Technology (Danvers, MA, USA). Monoclonal antibody against AdipoR1 (ab126611) was purchased from Abcam (Cambridge, UK). APC-conjugated anti-CD19 (115512), PE-conjugated anti-CD138 (142504), FITC-conjugated anti-rabbit IgG (406403), and anti-mouse CD16/32 antibody (101320) were purchased from BioLegend (San Diego, CA, USA). The ECL detection system was also purchased from Cell Signaling Technology. PVDF membranes were obtained from Millipore (Billerica, MA, USA). Akt1 siRNA (siAkt1), STAT3 siRNA (siSTAT3), control siRNA (siCTR), and fluorescein-conjugated control siRNA (FITC-siCTR) were purchased from Cell Signaling Technology. PI3K p110δ siRNA (siPI3K) was purchased from Santa Cruz Biotechnology (Dallas, TX, USA). The plasmid pCMV-HA-STAT3 was purchased from Bioworld (Nanjing, China). Entranster^TM^-R4000 was purchased from Engreen (Beijing, China). RPMI 1640 was purchased from Thermo Fisher Scientific (Waltham, MA, USA). Fetal bovine serum (FBS) was purchased from ScienCell Research Laboratories (Carlsbad, CA, USA). CD45R (B220) MicroBeads were obtained from Miltenyi Biotec (Aubum, CA, USA). Recombinant mouse AD was purchased from R&D Systems (Minneapolis, MN, USA). TRIzol was obtained from Invitrogen (Carlsbad, CA, USA). HiScript II Q RT SuperMix, 2× Taq Master Mix, and AceQ qPCR SYBR Green Master Mix were purchased from Vazyme (Nanjing, China). The reagent of eBioscience™ Fixable Viability Dye eFluor™ 450 was from Thermo Fisher Scientific (Waltham, MA, USA). Lipopolysaccharide (LPS) was from Sigma-Aldrich (St. Louis, MO, USA).

### Experimental Animals

Male DBA/1J and C57BL/6 mice aged 8–12 weeks were purchased from the Shanghai Laboratory Animal Center. Mice were fed and housed under specific pathogen-free conditions at the Experimental Animal Center of Nanjing Medical University. All experiments were conducted in compliance with the guidelines for the care and use of laboratory animals and approved by the Institutional Animal Care and Use Committee of Nanjing Medical University.

### Induction of CIA and Treatment With AD

Collagen-induced arthritis was induced by type II collagen (collagen II) immunization of DBA/1J mice, as previously described (13, 21). Mice with CIA were injected intraarticularly with AD [10 μg AD in 10 μl phosphate-buffered saline (PBS)] in their knee joints on days 17, 20, and 23 after the first immunization (13, 21). Control mice with CIA were injected with 10 μl PBS at the same time points. The mice were scored for joint inflammation everyday post the second immunization, with a maximum arthritis severity score of 16 for each mouse. The disease severity was evaluated by visually inspecting the paws of each mouse. Each paw was scored for the degree of inflammation on a scale from 0 to 4 as follows: 0, no evidence of erythema and swelling; 1, erythema and mild swelling confined to the midfoot (tarsals) or ankle joint; 2, erythema and mild swelling extending from the ankle to the midfoot; 3, erythema and mild swelling extending from the ankle to the metatarsal joints; 4, erythema and severe swelling encompassing the ankle, foot, and digits. The scores for each of the four paws were added to obtain the total score for each mouse. The incidence of CIA development was defined when arthritis severity score was ≥1.

### AdipoR1 Knockdown in Mice With CIA

Lentivirus-AdipoR1-shRNA (LV-shAdipoR1, 5′-GATTGCTCTACTGATTATG-3′) and lentivirus-control-shRNA (LV-shCTR, 5′-TTCTCCGAACGTGTCACGT-3′) were constructed by Genechem (Shanghai, China). LV-shAdipoR1 or LV-shCTR (1 × 10^6^) was injected intraarticularly into the knee joints of mice with CIA consecutively on day 21 following the first collagen II immunization. The mice were scored for joint inflammation everyday post the second immunization, as mentioned above.

### Cell Culture

Spleen naïve B cells from C57BL/6 mice were purified using CD45R (B220) MicroBeads according to the instructions of the manufacturer and the purity of B cells after B220 MicroBeads selection was determined by flow cytometry (Supplementary Figure 1). Purified B cells were cultured in RPMI 1640 supplemented with 10% (v/v) FBS and LPS (0.1 μg/ml) in the presence or absence of AD stimulation (0.1, 1, 10 μg/ml) at 37°C in 5% CO_2_.

### Cellular Transfection

The cultured purified B cells were transfected with siPI3K, siAkt1, siSTAT3, or siCTR using the Entranster^TM^-R4000 according to the instructions of the manufacturer. The transfection efficiency of siRNA was evaluated by the expression of FITC-siCTR in cells (Supplementary Figure 2).

### Flow Cytometry

The cultured purified mouse B cells or cells from mouse joint tissues were incubated with eBioscience™ Fixable Viability Dye eFluor™ 450 in PBS at 4°C for 30 min for eliminating dead cells. Next, cells were incubated with anti-mouse CD16/32 in PBS with 1% BSA at 4°C for 30 min for blocking FcR and other potential non-specific binding. Then, cells were stained by treating with different antibodies at 4°C for 30 min. After washing two times with PBS, the protein expression in cells was evaluated using flow cytometry with a BD FACSVerse (BD Biosciences, San Jose, CA, USA). The gating strategy of flow cytometry analysis was shown in Supplementary Figures 3, 4. In addition, for assessing proliferation with BrdU staining, cells were incubated with 10 μm of BrdU for the last 2 h before collection.

### Reverse Transcription Polymerase Chain Reaction (RT-PCR) and Quantitative Real-Time PCR (qPCR)

Total RNA was extracted from the synovium or cells using TRIzol reagent. For each sample, 1 μg RNA was reverse transcribed into cDNA using the HiScript II Q RT SuperMix according to the instructions of the manufacturer. PCR was performed using primers specific for mouse AdipoR1, Blimp-1, PAX-5, XBP-1, Bcl-6, and GAPDH mRNA in 2× Taq Master Mix according to the instructions of the manufacturer. The primer sequences are presented in Supplementary Table 1. The qPCR assay was conducted using AceQ qPCR SYBR Green Master Mix (Vazyme) in an ABI StepOnePlus and relative gene expression was calculated using the 2^−ΔΔCT^ method. The data were normalized to β-actin transcript expression.

### Western Blot Analysis

Cells were lysed using RIPA lysis buffer. Equal quantities of protein were separated by polyacrylamide gel electrophoresis in a 4–20% ExpressPlus™ PAGE Gel (Genscript, Nanjing, China). Western blot analysis was performed as described previously (22). The samples were treated with primary antibodies against β-actin, ERK1/2, p-ERK1/2, PI3K p110, Akt1, p-Akt1^Thr308^, p-Akt1^Ser473^, STAT3, p-STAT3, Egr-1, HRP-conjugated anti-rabbit IgG, and HRP-conjugated anti-mouse IgG to detect the expression of the corresponding proteins. The bands formed on the PVDF membranes were visualized using a GE Amersham Imager 600 with an ECL detection system. The densities of the radiographic bands were analyzed using Quantity One software (Bio-Rad, Hercules, CA, USA).

### Luciferase Reporter Assay

The luciferase reporter plasmids pGL3/Blimp-1-full-length (pGL3/Blimp-1-FL, −2000 ~ +200 nt), pGL3/Blimp-1-truncated (-1700 ~ +200, −1400 ~ +200, −1100 ~ +200, and −500 ~ +200), and pGL3/Blimp-1-FL-mutant (pGL3/Blimp-1-FL-M, −649 to −640 nt, from TTAATGGAAG to CCGGCTCTCT) were constructed by General Biosystems (Anhui, China). The activity of a full-length Blimp-1 gene promoter and its different truncated fragments or mutants in 293T cells or B cells was evaluated using a luciferase reporter assay, as mentioned previously (23).

### Chromatin Immunoprecipitation (ChIP)

Chromatin immunoprecipitation was performed using an antibody against STAT3 and preimmune IgG, as described previously (23). A proximal region in the Blimp-1 gene promoter (-719 ~ −523 nt) was amplified from the immunoprecipitated chromatin using PCR in 2× Taq Master Mix according to the instructions of the manufacturer. The primers used were as follows: sense, 5′-TGGACCCAGGAACGATG-3′; antisense, 5′-CCCTCTGGCGTCTGTCA-3′.

### ELISA

The culture supernatant of B cells *in vitro* was also gathered. IgG and IgM protein levels in the supernatant were determined by using the ELISA kits from Abcam (ab151276 and ab133047) according to the recommendations of the manufacturer.

### Histological Evaluation and Immunohistochemistry (IHC) Staining

The knee joints of mice with CIA were removed from the sacrificed mice for hematoxylin & eosin (H&E), Safranin O-fast green, and IHC analysis. Samples from each mouse were fixed in 4% buffered paraformaldehyde. Next, the tissues were cut into sections 3 μm in thickness, deparaffinized in xylene, and rehydrated by treating with a series of concentrations of ethanol. Tissue sections were prepared and stained with H&E or Safranin O-fast green. For IHC, heat-mediated antigen retrieval with sodium citrate buffer (pH6) was performed. After the inactivation of endogenous peroxidase, the sections were blocked by incubation in a 5% bovine serum album for 30 min at room temperature and then incubated overnight with rabbit anti-mouse IgG antibody (Abcam, ab6709, 1:1,000) at 4°C in a humidified chamber. After washing, the sections were incubated with HRP-conjugated anti-rabbit IgG (Abcam, ab6721, 1:1,000) for 1 h at room temperature. The reactions were developed using a DAB substrate kit. Each slide was evaluated by one of the authors under a microscope (Nikon, Tokyo, Japan). For histopathological evaluation, knee joint damage was scored under blinded conditions, using a widely used scoring system for the assessment of synovitis, cartilage degradation, and bone erosion (21).

### Statistical Analysis

Data are presented as mean ± SE. The Student's *t*-test or one-way ANOVA was performed to determine significant differences among groups. In cases where significant differences were found, individual comparisons were performed between groups using the *t*-statistic and adjusting the critical value according to the Bonferroni method. Differences with *p* < 0.05 were considered statistically significant.

## Results

### Local Intraarticular Injection of AD Induces B Cell Expansion in Joint Tissues and Exacerbates the Development of Arthritis in Mice With CIA

**Figure 1:**
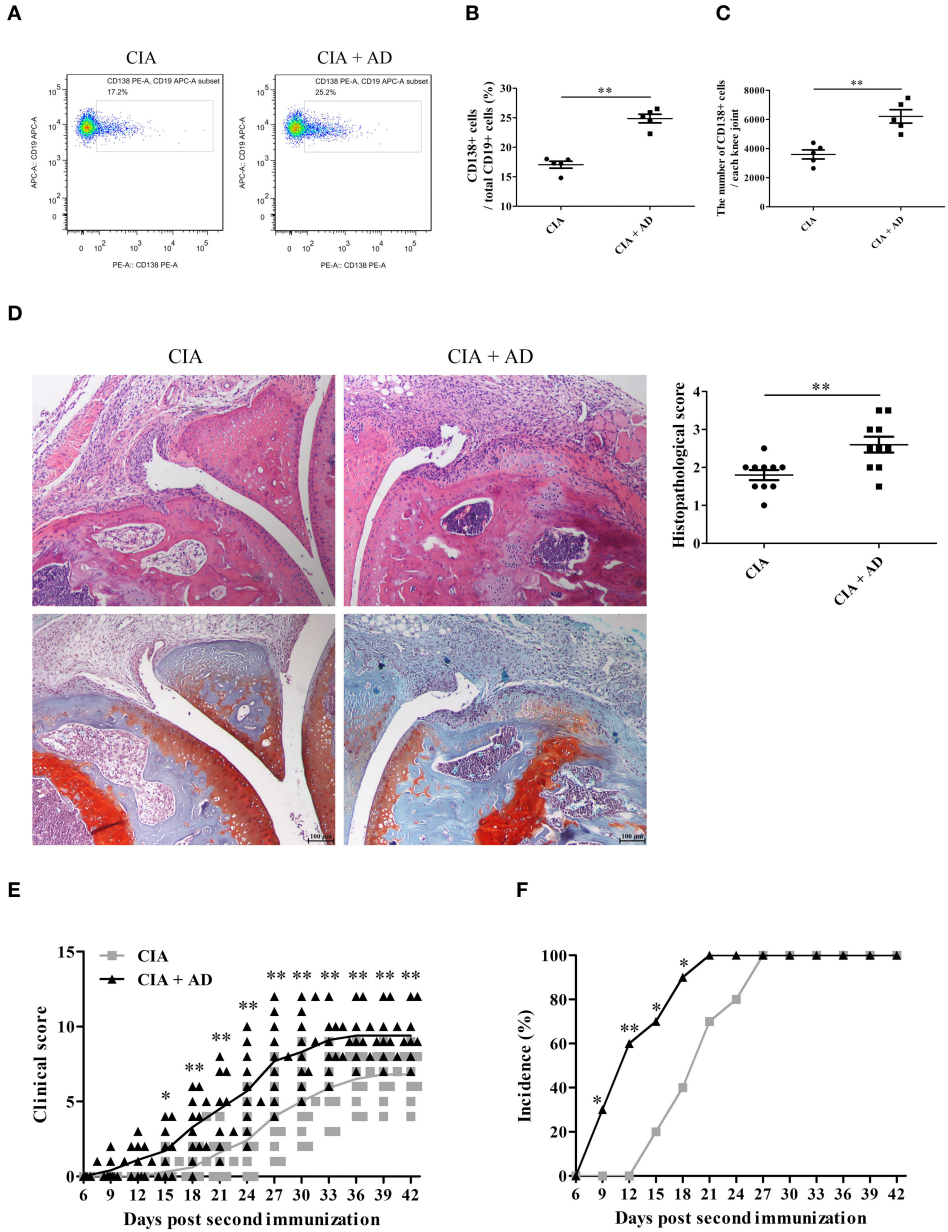
The effects of adiponectin (AD) on B-cell differentiation and arthritis of collagen-induced arthritis (CIA) mice. AD was injected into the knee joints of mice with CIA. **(A–C)** CD138 expression on B cells from the joint tissues of CIA mice with or without AD treatment on day 35 post the first collagen II immunization was detected by flow cytometry, and CD138-positive cell percentage **(B)** and count **(C)** were expressed, respectively. **(D)** Histopathologic evaluation of hematoxylin & eosin (H&E) and Safranin O-fast green stained knee joint tissue with assessment on the scores of joint damage. **(E,F)** The arthritis scores **(E)** and incidence **(F)** were evaluated in CIA mice with or without AD treatment. Results from one representative experiment out of three were shown. Representative images are shown. Data are presented as means ± SE (*n* = 10 in each group). *p < 0.05; **p < 0.01.

In this study, we first examined whether AD could induce the differentiation of B cells to plasma cells in joint tissues when locally injected into the knee joints of mice with CIA. A dose of 10 μg of AD was injected intraarticularly into the knee joints of mice with CIA on days 17, 20, and 23 after the 1st collagen II immunization (13, 21). We observed that the plasma cell count increased after the local intraarticular AD injection (Figures 1A–C). Moreover, histopathological examination with H&E and Safranin O-fast green staining of the knee joint samples showed significantly increased synovial hyperplasia, cartilage damage, and bone erosion in AD-treated mice with CIA when compared with untreated control CIA mice (Figure 1D). As shown in Figures 1E,F, AD-treated mice with CIA exhibited an earlier onset of arthritis and higher arthritis scores than the untreated control CIA mice. Collectively, the locally increased levels of AD in the joints of mice with CIA induced plasma cell expansion and exacerbated the signs of arthritis, suggesting that AD might participate in disease progression in mice with CIA by promoting B cell differentiation.

### Knockdown of AdipoR1 Reduces Plasma Cell Differentiation and Arthritic Damage in CIA Mice

**Figure 2:**
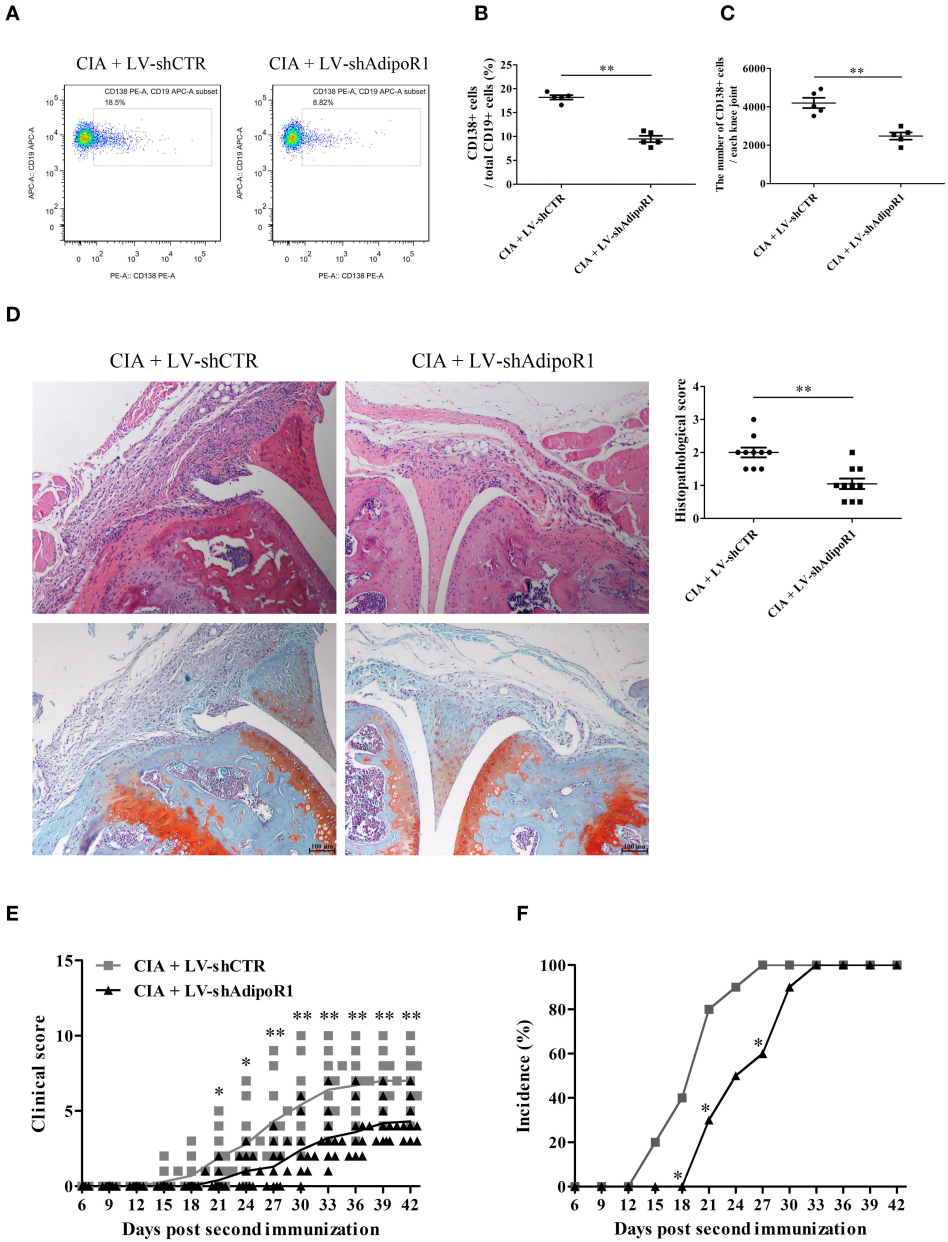
The effects of AdipoR1 knockdown on plasma cell differentiation and arthritic damage of CIA mice. LV-shAdipoR1 and LV-shCTR were intraarticularly injected into the knee joints of CIA mice consecutively on days 21 post the first collagen II immunization. **(A–C)** CD138 expression on B cells from the joint tissues of mice on day 35 post the first collagen II immunization was detected by flow cytometry, and CD138-positive cell percentage **(B)** and count **(C)** were expressed, respectively. **(D–F)** The histopathologic analysis with H&E and Safranin O-fast green staining **(D)**, arthritis scores, **(E)** and incidence **(F)** were evaluated in mice. Results from one representative experiment out of three are shown. Representative images are shown. Data are presented as means ± SE (*n* = 10 in each group). *p < 0.05; **p < 0.01.

To confirm whether AD modulates B cell differentiation, LV-shAdipoR1 was used to induce AdipoR1 knockdown in the knee joints. LV-shAdipoR1 and LV-shCTR were intraarticularly injected into the knee joints of CIA mice consecutively on day 21 after the first collagen II immunization. We also observed that plasma cells were decreased in response to the local intraarticular injection of LV-shAdipoR1 for silencing AdipoR1 expression (Supplementary Figure 5, Figures 2A–C). As shown in Figure 2D and Supplementary Figure 6, pronounced synovial hyperplasia, cartilage damage, bone erosion, and IgG deposition were observed in the knee joint tissues of the mice from the CIA group, whereas only mild synovial inflammation and tissue damage with limited IgG deposition were observed in the knee joint of mice from the shAdipoR1-treated group. More importantly, shAdipoR1-treated mice with CIA exhibited a later onset of arthritis and lower arthritis scores than the LV-shCTR-treated control mice with CIA (Figures 2E,F).

### Adiponectin Promotes B Cell Proliferation and Differentiation *in vitro*

**Figure 3:**
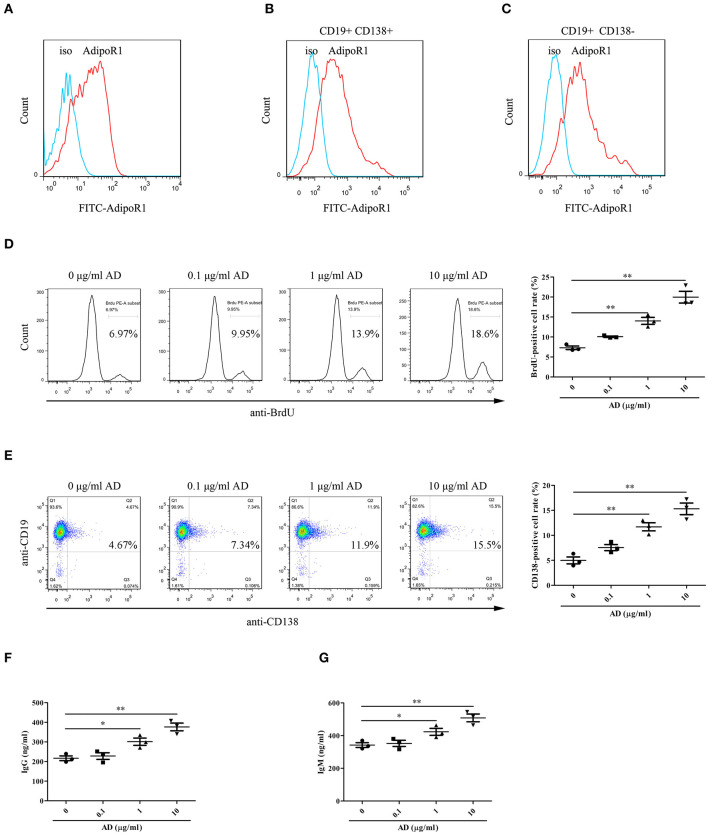
The roles of AD in B-cell proliferation and differentiation *in vitro*. **(A)** The expression of AdipoR1 on purified mouse splenic B cells from normal mice was detected by flow cytometry. **(B,C)** The expression of AdipoR1 on CD19^+^CD138^+^ B cells **(B)** and CD19^+^CD138^−^ B cells **(C)** in knee joints of CIA mice was detected by flow cytometry. **(D,E)** Purified mouse splenic B cells were incubated with different doses of AD, and the BrdU-positive cell rate (**(D)**, 72 h) and CD138-positive cell rate (**(E)**, 96 h) were detected by flow cytometry. **(F,G)** The levels of IgG **(F)** and IgM **(G)** were obviously elevated in the supernatant of AD-treated B cells by ELISA. Results from one representative experiment out of three are shown. Representative images are shown. Data are presented as means ± SE (*n* = 3 in each group). *p < 0.05, **p < 0.01.

To confirm the possible effects of AD on B cell proliferation and differentiation, the expression of AD receptor in splenic B cells from normal mice was first evaluated. The results indicated that the splenic B cells expressed high levels of the AD receptor AdipoR1 (Figure 3A). Notably, B cells from knee joints of CIA mice also expressed AdipoR1 was determined (Figures 3B,C). Purified splenic B cells were stimulated with different doses of AD and cultured for 72 and 96 h for assessing proliferation and differentiation, respectively. We observed that AD stimulation *in vitro* markedly enhanced B cell proliferation (Figure 3D) and differentiation into plasma cells (Figure 3E) in a dose-dependent manner. Consistently, the levels of IgG/IgM were significantly elevated in the supernatant of AD-treated B cell cultures (Figures 3F,G). Collectively, these results indicated that AD could directly promote B cell proliferation and differentiation.

### Adiponectin Induces Blimp-1 Expression and Akt1/STAT3 Activation in B Cells

**Figure 4:**
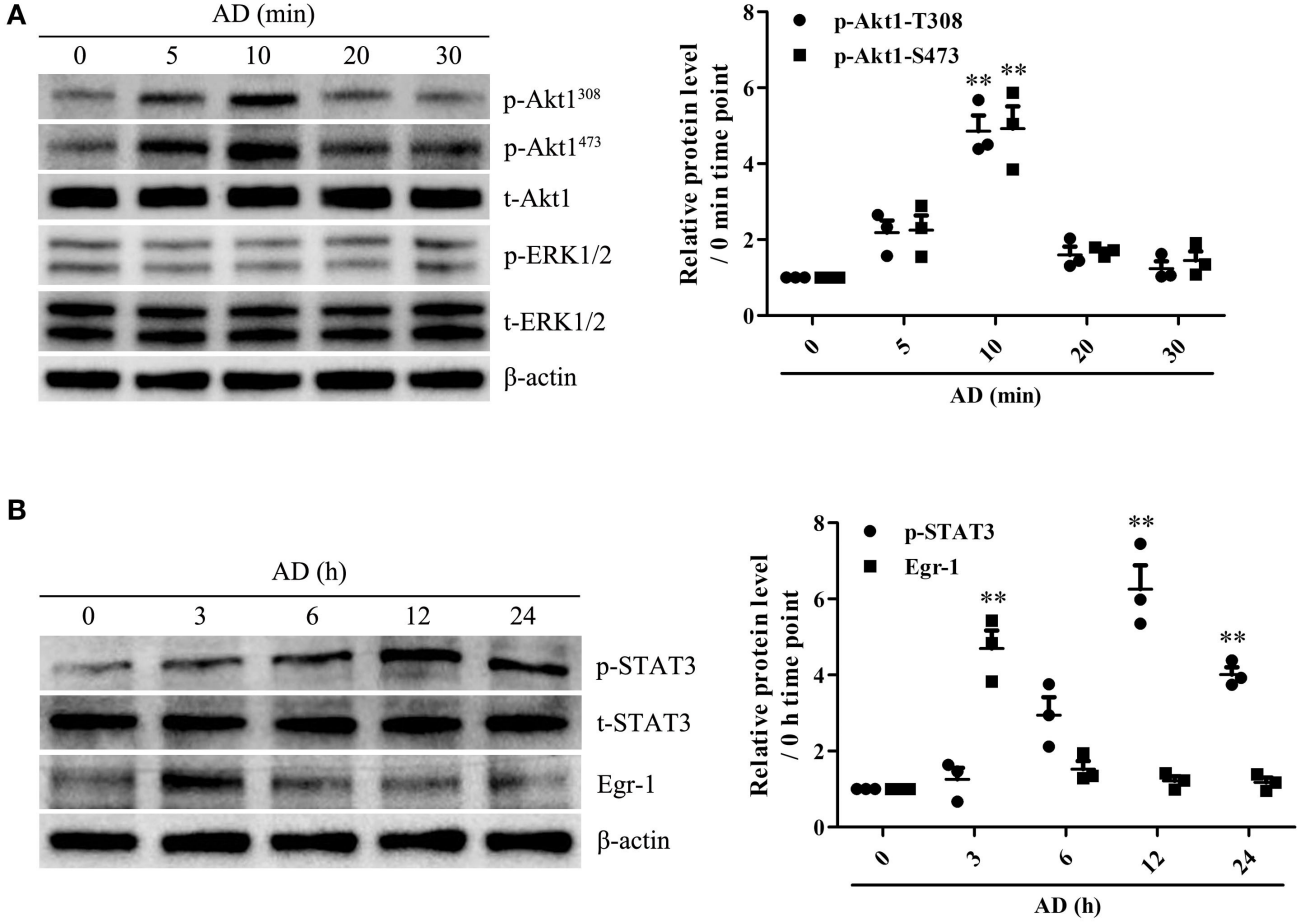
The expression and activation of Akt1, ERK1/2, Egr-1, and STAT3 in B cells exposed to AD. **(A)** Purified mouse splenic B cells were incubated with 10 μg/ml AD for different time points (0, 5, 10, 20, 30 min), and the levels of t-Akt1, p-Akt1^Thr308^, p-Akt1^Ser473^, t-ERK1/2, p-ERK1/2, and β-actin were examined by the Western blot. **(B)** Purified mouse splenic B cells were incubated with 10 μg/ml AD for different time points (0, 3, 6, 12, and 24 h), and the levels of Egr-1, t-STAT3, p-STAT3, and β-actin were detected by the Western blot. Results from one representative experiment out of three are shown. Representative images are shown. Data are presented as means ± SE (*n* = 3 in each time points). **p < 0.01 vs. 0 min time point or 0 h time point.

To identify the potential genes encoding proteins associated with B-cell proliferation and differentiation in response to AD stimulation, the mRNA expression levels of Blimp-1, PAX-5, XBP-1, and Bcl-6 in B cells were measured using RT-PCR. We observed that AD treatment markedly upregulated Blimp-1 expression and downregulated PAX-5 expression in B cells, whereas, it did not exert any significant effect on XBP-1 and Bcl-6 expression (Supplementary Figure 7), which indicates that Blimp-1 might be involved in AD-induced B-cell differentiation. Recently, AD was reported to induce the activation of intracellular signaling molecules such as Akt1 and ERK1/2 (18, 19) and transcriptional factors such as Egr-1 and STAT3 (20, 24). We evaluated the effects of AD on the expression and activation of the abovementioned molecules. The results showed that AD stimulation significantly promoted the phosphorylation of Akt1 and STAT3, whereas it did not promote the phosphorylation of ERK1/2 in purified B cells (Figure 4). Although Egr-1 expression increased in purified B cells after AD stimulation, the time point of occurrence was considerably earlier than that for Blimp-1. Based on this, we continued to explore the possible role of STAT3 in Blimp-1 expression.

### PI3K/Akt1/STAT3 Activation Contributes to B-Cell Proliferation and Differentiation

**Figure 5:**
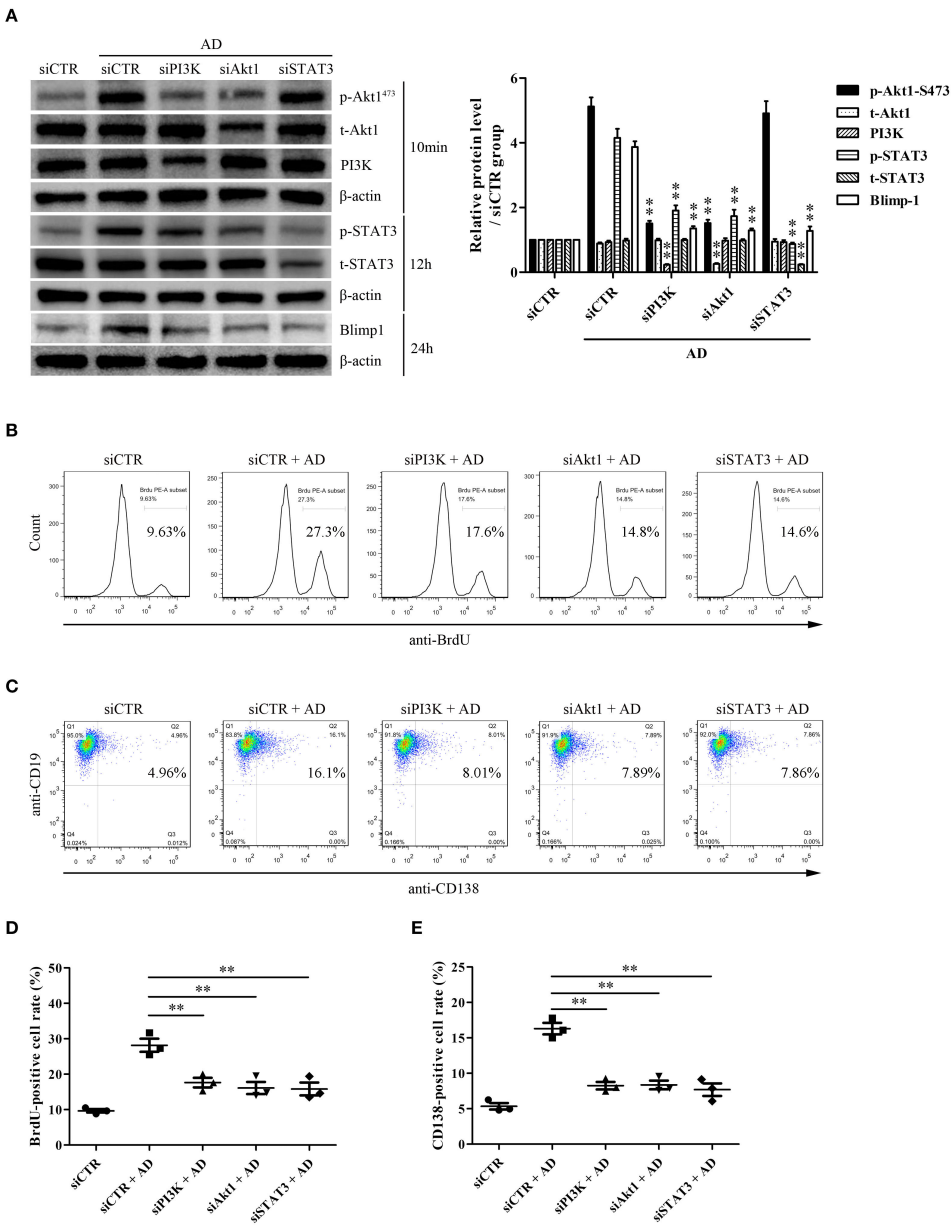
The roles of PI3K, Akt1, and STAT3 activation in B-cell proliferation and differentiation in response to AD. Purified mouse splenic B cells were transfected with siPI3K, siAkt1, siSTAT3, or siCTR followed by AD stimulation (10 μg/ml) for different time points, and then the protein expression as well as B-cell proliferation and differentiation were evaluated. **(A)** The levels of PI3K, t-Akt1, p-Akt1^Ser473^, t-STAT3, p-STAT3, Blimp-1, and β-actin were examined by the Western blot. **(B–E)** The BrdU-positive rate **(B,D)** and CD138-positive rate **(C,E)** were detected by flow cytometry. Results from one representative experiment out of three are shown. Representative images are shown. Data are presented as means ± SE (*n* = 3 in each group). **p < 0.01 vs. siCTR + AD group.

To evaluate the possible roles of PI3K/Akt1 and STAT3 in Blimp-1 expression and AD-induced B-cell proliferation and differentiation, purified B cells were transfected with siPI3K, siAkt1, siSTAT3, or siCTR followed by AD stimulation, following which B-cell proliferation and differentiation were evaluated. The results showed that PI3K knockdown inhibited Akt1 and STAT3 phosphorylation, and Akt1 knockdown inhibited STAT3 phosphorylation; however, STAT3 knockdown did not exert any significant effect on Akt1 phosphorylation (Figure 5A), which confirmed the upstream and downstream relationships among PI3K, Akt1, and STAT3. In addition, PI3K, Akt1, and STAT3 knockdown significantly inhibited Blimp-1 expression (Figure 5A) and B-cell proliferation (Figures 5B,D) and differentiation (Figures 5C,E). These data indicate that the activation of Akt1 and STAT3 contributes to AD-induced B-cell proliferation and differentiation.

### Adiponectin-Mediated STAT3 Activation Regulates the Transcription of Blimp-1 Gene in B Cells

**Figure 6:**
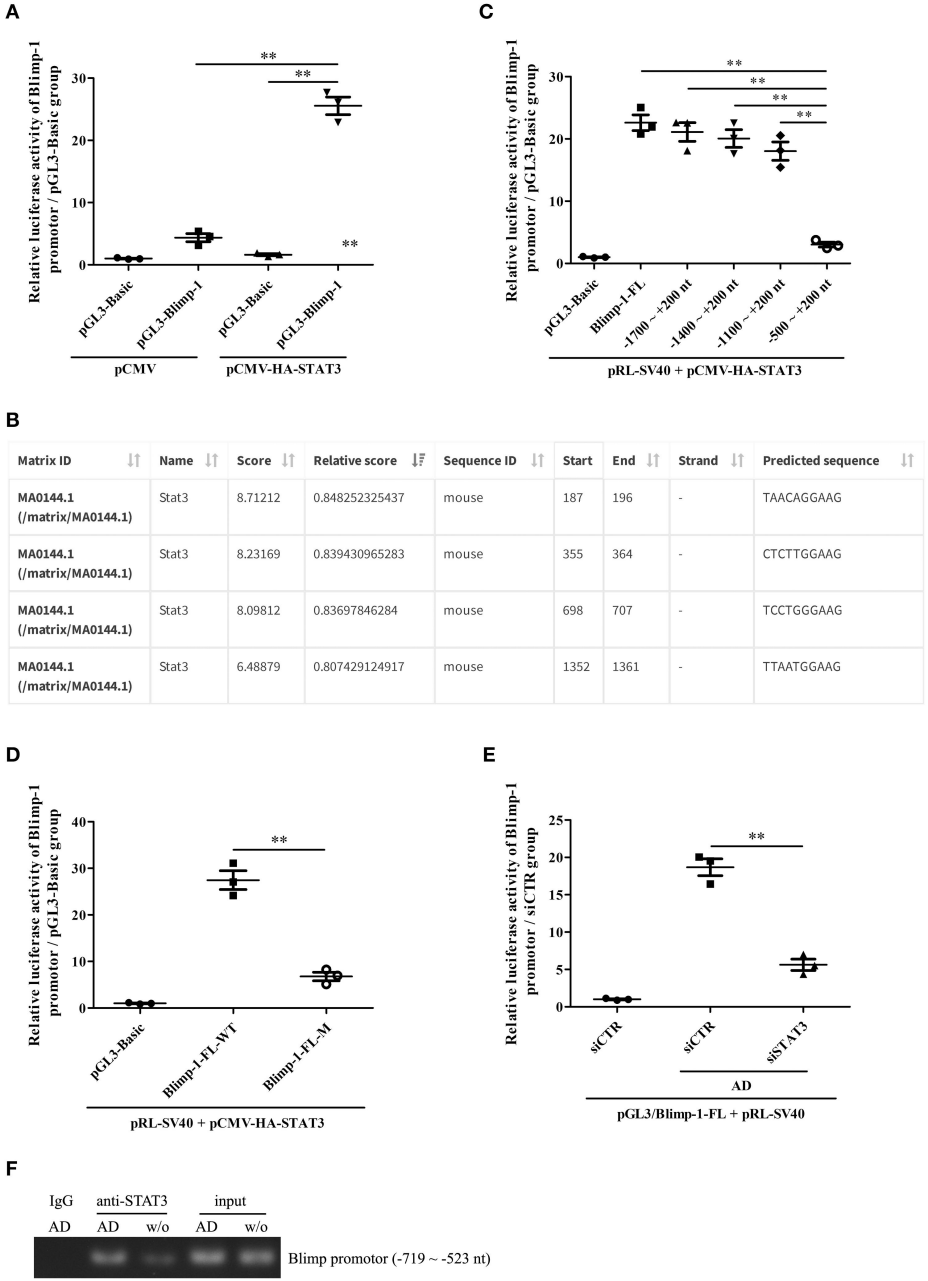
The regulation of STAT3 activation on Blimp-1 gene transcription. **(A)** The luciferase reporters of mouse Blimp-1 gene promoter full-length (pGL3-Blimp-1), pRL-SV40, and pCMV-HA-STAT3 or pCMV were co-transfected into 293T cells, the luciferase activity was detected. **(B)** The STAT3-binding elements within mouse Blimp-1 gene promoter were predicted by JASPAR. **(C)** The plasmids of full-length (Blimp-1-FL) or different truncated Blimp-1 gene promoter (−1,700 ~ +200 nt, −1400 ~ +200 nt, −1,100 ~ +200 nt, −500 ~ +200 nt), pRL-SV40, and pCMV-HA-STAT3 were co-transfected into 293T cells, the luciferase activity was determined. **(D)** The plasmids of wild-type full-length Blimp-1 gene promoter (Blimp-1-FL-WT) or mutated full-length Blimp-1 gene promoter (−649 ~ −640 nt, Blimp-1-FL-M), pRL-SV40, and pCMV-HA-STAT3 were co-transfected into 293T cells, the luciferase activity was detected. **(E)** The plasmids of pGL3-Blimp-1-FL, pRL-SV40, and siSTAT3 or siCTR were co-transfected into purified mouse splenic B cells followed by AD stimulation, and then the luciferase activity was examined. **(F)** Chromatin immunoprecipitation (ChIP) was used to pull-down STAT3-DNA complexes from purified mouse splenic B cells stimulated with or without AD by using an anti-STAT3 antibody or IgG control, and then PCR was performed to amplify the DNA sequence of −719 ~ −523 nt. Results from one representative experiment out of three are shown. Data are presented as means ± SE (*n* = 3 in each group). **p < 0.01.

We further explored the possible mechanism underlying STAT3-mediated Blimp-1 gene transcription in B cells. We first constructed luciferase reporters of the full-length Blimp-1 gene promoter (FL, namely, −2000 ~ +200 nt). The luciferase reporter assays showed that the activity of the Blimp-1 gene promoter increased significantly compared to that in the pCMV-HA-STAT3-transfected 293T cells, suggesting that STAT3 enhanced Blimp-1 gene transcription (Figure 6A). We further constructed luciferase reporters of different depleted Blimp-1 gene promoter fragments (−1,700 to +200 nt, −1,400 to +200 nt, −1,100 ~ +200 nt, −500 ~ +200 nt) based on JASPAR prediction (Figure 6B). The luciferase reporter assays showed that the activity of the Blimp-1 gene promoter (−500 to +200 nt) decreased observably compared to that observed when FL and other depleted fragments were used with the pCMV-HA-STAT3-transfected 293T cells (Figure 6C), indicating that the −1100 to −500 nt region might contain an important STAT3-binding element. Subsequent experiments showed that the mutation of the STAT3-binding element at −649 to −640 nt predicted by JASPAR within the −1100 to −500 nt region could significantly inhibit the luciferase activity of Blimp-1 gene promoter in pCMV-HA-STAT3-transfected 293T cells (Figure 6D). The siRNA-mediated knockdown of STAT3 inhibited the luciferase activity of the Blimp-1 gene promoter in B cells stimulated with AD (Figure 6E). In addition, ChIP experiments showed that AD stimulation could increase the binding of STAT3 to the −719 to −523nt DNA sequence of Blimp-1 gene promoter in the B cells (Figure 6F). Collectively, these data indicate that STAT3 regulated Blimp-1 gene transcription in B cells by binding to its promoter.

## Discussion

Increasing evidence indicates the roles of adipose tissue-derived adipokines such as AD and leptin in RA pathogenesis (10, 12). In our previous studies, we have shown that the expression levels of AD are high in the inflamed joint synovial tissues, with elevated levels observed in the synovial fluid collected from patients with RA (14). Moreover, recombinant AD was observed to stimulate the production of monocyte chemoattractant protein-1 and interleukin-6 in cultured synovial fibroblasts, which indicates the plausible role of AD in synovial inflammation and progressive bone erosion in patients with RA (14). Although AD has been shown to exert diverse functions, such as promoting T cell responses, synovial inflammation, and joint tissue damage in RA, the effects of AD on B-cell proliferation and differentiation during CIA development remain unclear (13). In this study, we first show that local intraarticular injection of AD promoted an earlier arthritic onset and exacerbated arthritis development in CIA mice. Importantly, we have observed an increased number of plasma cells in the joint tissue of CIA mice with intraarticular injection of AD while local injection of LV-shAdipoR1 to knockdown AdipoR1 expression resulted in a reduced number of plasma cells in the knee joints. Although it remains unclear whether increased plasma cells in joint tissues result from enhanced survival or increased B-cell differentiation *in situ*, our further studies reveal that AD promotes both proliferation and differentiation of B cells in culture. Moreover, splenic B cells and B cells purified from joint tissues of CIA mice express high levels of the AD receptor AdipoR1. Together, these data reveal a previously unrecognized role of AD in enhancing B-cell response and driving autoimmune inflammation during CIA development. Our experimental data show that articular AdipoR1 knockdown reduces IgG production in the knee joints of CIA mice, suggesting that local AD may stimulate B cell activation and differentiation into IgG-secreting plasma cells in the knee joint to produce anti-collagen antibodies, which may represent a mechanism by which AD drives B-cell response in addition to its recognized function in promoting T-cell response, leading to aggravated arthritis progression during the pathogenesis of CIA. Interestingly, Lee et al. (25) used AD-targeting antibodies to ameliorate rheumatic symptoms in mice with CIA, which might serve as a potential therapeutic strategy in the treatment of patients with RA. However, several studies (26, 27) reported that treatment with AD resulted in significantly delayed onset of arthritis as well as decreased clinical arthritis and histopathological severity scores. In view of both anti- and pro-inflammatory effects exerted by AD under different conditions *in vivo*, more studies are needed to explore AD-related therapeutic strategies for the treatment of RA.

It has been reported AD activates PI3K/Akt1 and STAT3 in certain types of cells, such as the epithelial cells, the endothelial cells, and the liver cancer cells (18–20). Additionally, both PI3K/Akt1 activation and STAT3 activation were shown to play pivotal roles in B-cell proliferation and differentiation (15, 16). The findings from this study indicated that AD could induce Akt1 and STAT3 activation in purified mouse B cells *in vi tro*. Furthermore, either PI3K/Akt1 or STAT3 activation contributed to B-cell proliferation and differentiation, in which PI3K/Akt1 upregulated STAT3 activation as upstream regulators of STAT3. Further studies are warranted to evaluate the possible mechanism underlying the upregulation of STAT3 *via* the activation of PI3K/Akt1 in the B cells stimulated with AD.

Blimp-1, encoded by the PRDM1 gene, is a member of the PRDM family of transcription repressors that controls B-cell differentiation. The regulation of Blimp-1 expression has been studied extensively in lymphocytes, which involves the contribution of transcription factors such as activator protein 1 (AP1), interferon regulatory factor-4 (IRF-4), and STAT3 (17, 28, 29). For example, Sean et al. (17) have reported that STAT3-mediated upregulation of Blimp-1 is coordinated with BCL6 downregulation to control human B-cell differentiation to plasma cells. The present study showed that AD-induced PI3K/Akt1/STAT3 activation upregulated Blimp-1 expression and promoted B-cell differentiation, in which PI3K/Akt1-activated STAT3 bound to the Blimp-1 gene promoter and induced transcription, indicating that the activation of the PI3K/Akt1/STAT3 axis could play an important role in regulating B-cell differentiation in response to AD stimulation. In addition to Blimp-1 gene, the regulation of other target genes might be mediated by the activation of the PI3K/Akt1/STAT3 axis in the B cells, which might contribute to AD/PI3K/Akt1/STAT3-induced B-cell proliferation.

Our findings demonstrate that AD exacerbates CIA by enhancing B-cell proliferation and differentiation *via* the PI3K/Akt1/STAT3 axis; these findings may provide novel insights into the role of B cells in RA pathogenesis.

## Data Availability Statement

The raw data supporting the conclusions of this article will be made available by the authors, without undue reservation.

## Ethics Statement

The animal study was reviewed and approved by Institutional Animal Care and Use Committee of Nanjing Medical University.

## Author Contributions

WQ, MZ, and LL designed and wrote the manuscript. NC, XS, LG, XW, JS, YS, LX, RL, JW, FZ, NP, and WQ performed the experiments. NC, XS, WQ, FX, and DH analyzed the data. All authors contributed to the article and approved the submitted version.

## Conflict of Interest

The authors declare that the research was conducted in the absence of any commercial or financial relationships that could be construed as a potential conflict of interest.
