# Supplementary material for: Adiponectin Enhances B-Cell Proliferation and Differentiation via Activation of Akt1/STAT3 and Exacerbates Collagen-Induced Arthritis
Source: Front Immunol. 2021 Mar 18;12:626310. doi: 10.3389/fimmu.2021.626310 (PMC8012765; doi:10.3389/fimmu.2021.626310)
Supplement: Supplementary file 1 [file Data_Sheet_1.docx]

**Supplemental Table 1**

|  | forward primer (5’→3’) | reverse primer (5’→3’) |
| --- | --- | --- |
| AdipoR1  Blimp-1 | AAGATCAAGCATGCCCGGTG  CGTGAAGTTTCAAGGACTGGC | CTCTGTGTGGATGCGGAAGA  TGGTGGAACTCCTCTCTGGA |
| PAX-5 | AAGGTTGCCACTCCCAAAGT | GTTTGTTGGTGTCGGCACTG |
| XBP-1 | AACAGAGTAGCAGCGCAGA | CCACCTCTGGAACCTCGTCA |
| Bcl-6 | CATCATGGCCTACCGAGGTC | GCTCGAAATGCAGGGCAATC |
| GAPDH | CCGGTGCTGAGTATGTCGTG | TACTTGGCAGGTTTCTCCAGG |

**Supplemental Table 1 The primer sequences of target genes.** The sequences of primers against AdipoR1, Blimp-1, PAX-5, XBP-1, Bcl-6 and GAPDH mRNA were listed.

**Supplemental Figure 1**

**
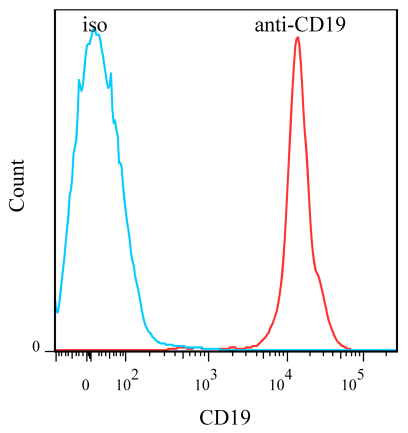
**

**Supplemental Figure 1. The purity of B cells after B220 MicroBeads selection.**

The purity of B cells after B220 MicroBeads selection was determined by flow cytometry with APC-conjugated anti-CD19 antibody and APC-conjugated isotype control antibody. Representative images are shown.

**Supplemental Figure 2**

non-conjugated siCTR FITC-conjugated siCTR

**



**

**Supplemental Figure 2. The transfection efficiency of B cells *in vitro*.**

Purified spleen B cells were transfected with FITC-conjugated siCTR or non-conjugated siCTR, and the flow cytometry was performed to detect FITC expression in cells. Representative images are shown.

**Supplemental Figure 3**

**A B**

**
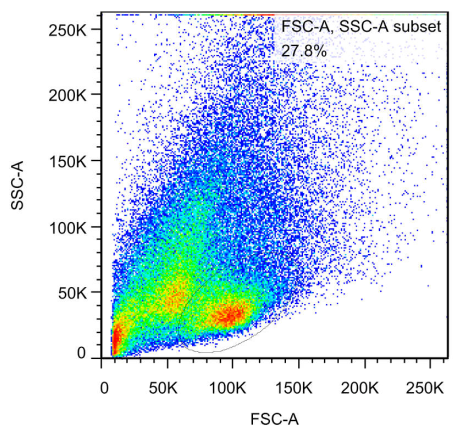

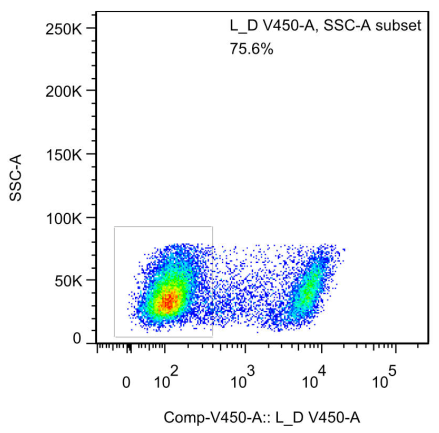
**

**C D**

**
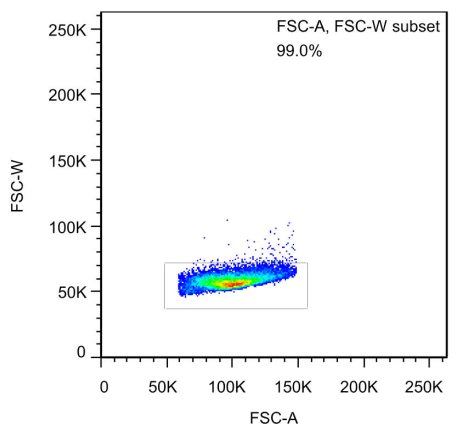

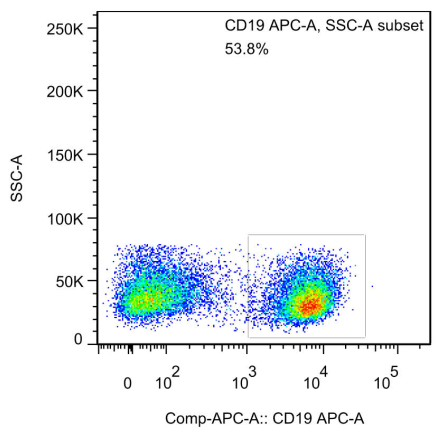
**

**Supplemental Figure 3. Flow cytometry gating procedure *in vivo*.**

Flow cytometry gating methods *in vivo* were shown. A: Lymphocytes gating; B: Living cells gating with eBioscience™ Fixable Viability Dye eFluor™ 450 negative staining; C: Single cells gating with FSC-A and FSC-W; D: CD19-positive cells gating with APC-conjugated anti-CD19 antibody staining. 10000 gated cells were further analyzed.

**Supplemental Figure 4**

**A B**

**
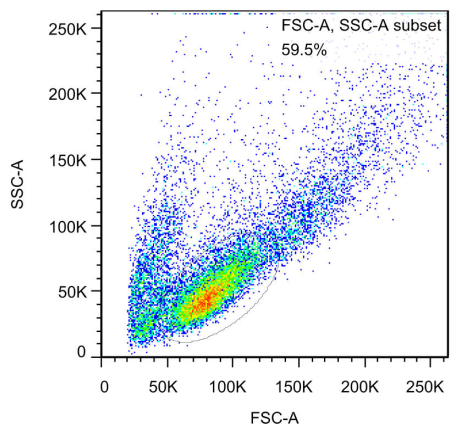

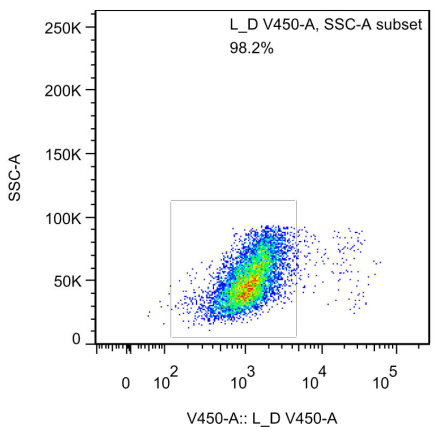
**

**C D**

**
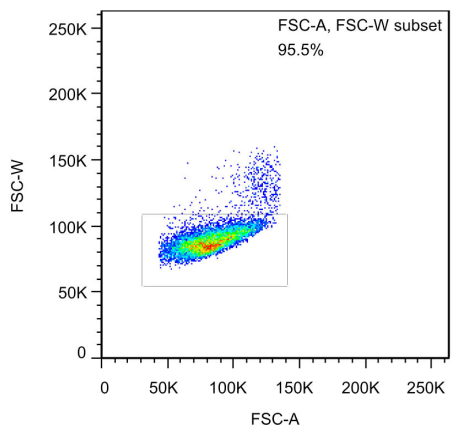

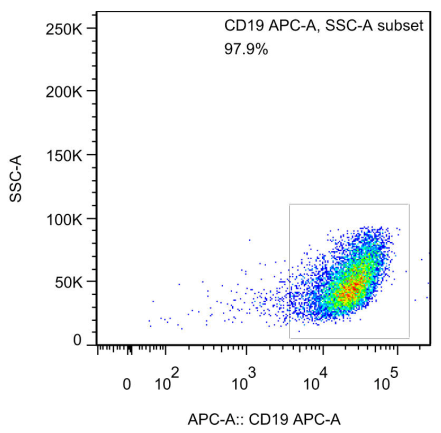
**

**Supplemental Figure 4. Flow cytometry gating procedure *in vitro*.**

Flow cytometry gating methods *in vitro* were shown. A: Lymphocytes gating; B: Living cells gating with eBioscience™ Fixable Viability Dye eFluor™ 450 negative staining; C: Single cells gating with FSC-A and FSC-W; D: CD19-positive cells gating with APC-conjugated anti-CD19 antibody staining. 10, 000 gated cells were further analyzed.

**Supplemental Figure 5**

**
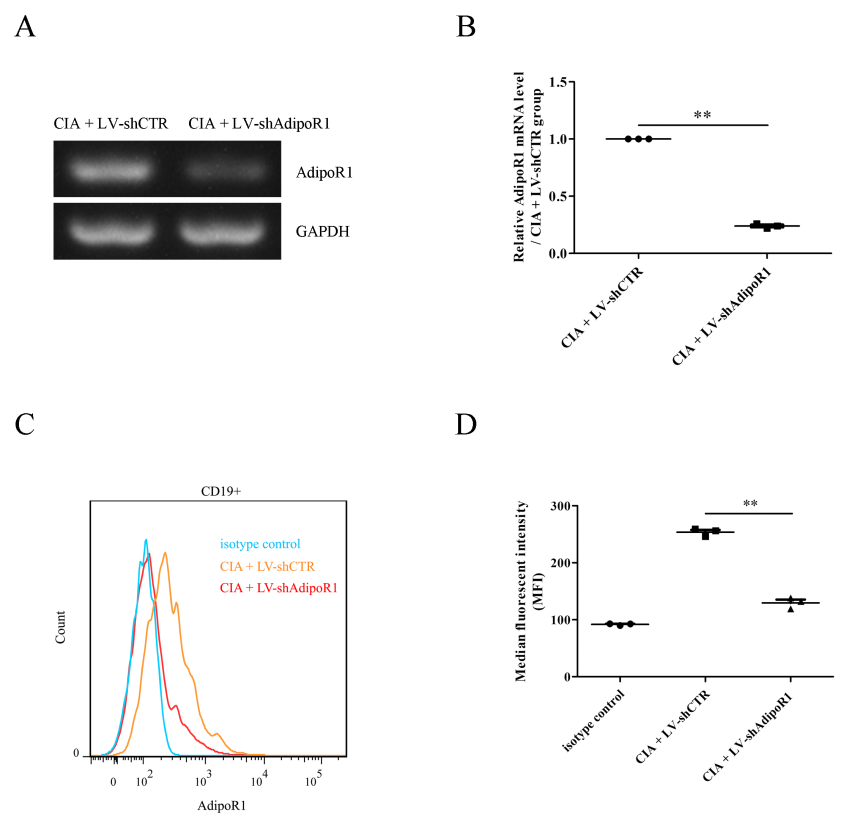
**

**Supplemental Figure 5. The effect of LV-shAdipoR1 on AdipoR1 expression in the knee joint synovium of CIA mice.** A and B: RT-PCR was done to evaluate the mRNA levels of AdipoR1 and GAPDH in the knee joint synovium of mice from CIA + LV-shCTR group and CIA + LV-shAdipoR1 group. C and D: Flow cytometry was performed to detect the expression of AdipoR1 on CD19-positive cells in the knee joint synovium of mice from CIA + LV-shCTR group and CIA + LV-shAdipoR1 group. Median of fluorescent intensity (MFI) AdipoR1 expression was expressed. Results from one representative experiment out of three were shown. Representative images are shown. Data are presented as means ± SE (*n* = 3 in each group). ** *P* < 0.01 vs. LV-shCTR.

**Supplemental Figure 6**

**CIA + LV-shCTR CIA + LV-shAdipoR1**

**
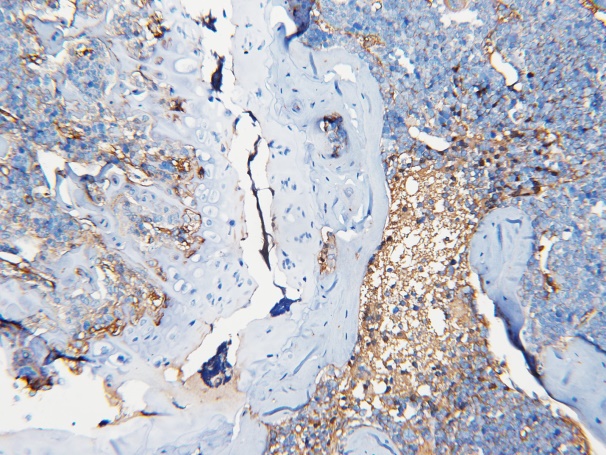

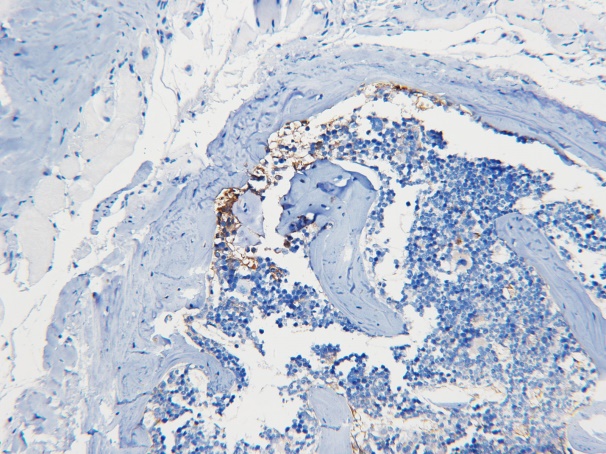
**

**Supplemental Figure 6. The effect of AdipoR1 knockdown on IgG deposition in the knee joints of CIA mice.** IHC staining for IgG was performed in the knee joint sections of mice from CIA + LV-shCTR group and CIA + LV-shAdipoR1 group. Results from one representative experiment out of three were shown. Representative images are shown (n = 10 in each group).

**Supplemental Figure 7**

**
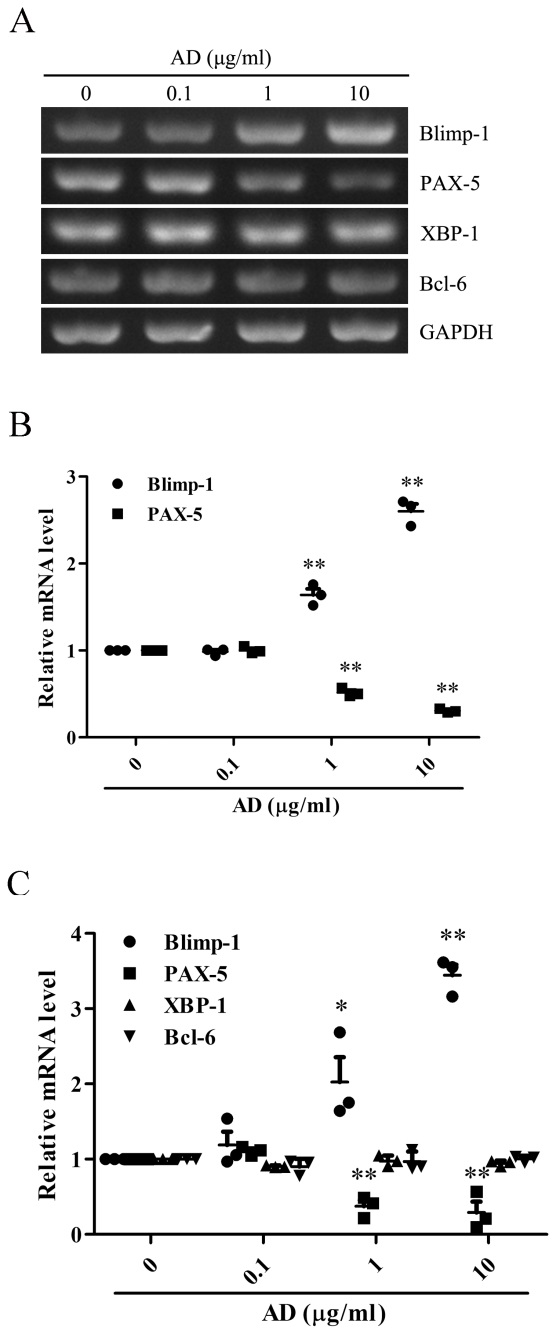
**

**Supplemental Figure 7. The effects of AD on Blimp-1, PAX-5, XBP-1 and Bcl-6 expression in B cells.** Purified mouse splenic B cells were incubated with different doses of AD, and the mRNA expression of Blimp-1, PAX-5, XBP-1, Bcl-6 and GAPDH in B cells at 24h was examined by RT-PCR (A and B) and qPCR (C). Results from one representative experiment out of three were shown. Representative images are shown. Data are presented as means ± SE (*n* = 3 in each group). * *P* < 0.05; ** *P* < 0.01 vs. 0 μg/ml dose.
